# Mobility, Balance and Falls in Persons with Multiple Sclerosis

**DOI:** 10.1371/journal.pone.0028021

**Published:** 2011-11-22

**Authors:** Jacob J. Sosnoff, Michael J. Socie, Morgan K. Boes, Brian M. Sandroff, John H. Pula, Yoojin Suh, Madeline Weikert, Swathi Balantrapu, Steven Morrison, Robert W. Motl

**Affiliations:** 1 Department of Kinesiology and Community Health, University of Illinois at Urbana-Champaign, Urbana, Illinois, United States of America; 2 Department of Mechanical Science and Engineering, University of Illinois at Urbana-Champaign, Urban, Illinois, United States of America; 3 Department of Bioengineering, University of Illinois at Urbana-Champaign, Urbana, Illinois, United States of America; 4 University of Illinois College of Medicine at Peoria, Peoria, Illinois, United States of America; 5 School of Physical Therapy, Old Dominion University, Norfolk, Virginia, United States of America; Oslo University Hospital, Norway

## Abstract

**Background:**

There is a lack of information concerning the relation between objective measures of gait and balance and fall history in persons with MS (PwMS). This investigation assessed the relation between demographic, clinical, mobility and balance metrics and falls history in persons with multiple sclerosis (MS).

**Methods:**

52 ambulatory persons with MS (PwMS) participated in the investigation. All persons provided demographic information including fall history over the last 12 months. Disease status was assessed with Expanded Disability Status Scale (EDSS). Walking speed, coordination, endurance and postural control were quantified with a multidimensional mobility battery.

**Results:**

Over 51% of the participants fell in the previous year with 79% of these people being suffering recurrent falls. Overall, fallers were older, had a greater prevalence of assistive devices use, worse disability, decreased walking endurance, and greater postural sway velocity with eyes closed compared to non-fallers. Additionally, fallers had greater impairment in cerebellar, sensory, pyramidal, and bladder/bowel subscales of the EDSS.

**Conclusions:**

The current observations suggest that PwMS who are older, more disabled, utilize an assistive device, have decreased walking coordination and endurance and have diminished balance have fallen in the previous year. This suggests that individuals who meet these criteria need to be carefully monitored for future falls. Future research is needed to determine a prospective model of falls specific to PwMS. Additionally, the utility of interventions aimed at reducing falls and fall risk in PwMS needs to be established.

## Introduction

Over 50% of persons with multiple sclerosis (PwMS) report falling over a 6-month period [1], [2], [3] and routinely require medical attention for fall-related injuries [3], [4], [5]. Additionally, the impact of a fall extends past the actual adverse event as a fall can result in activity curtailment, physiological deconditioning, and institutionalization [6]. Despite the adverse impact that falls can have on PwMS, there have been few direct investigations of the factors that contribute to falls in this high risk population. Such investigations indicate that poor balance, worse disability status, and use of assistive device are related to fall history in PwMS [1], [2], [3], [7].

Although previous investigations have provided important information concerning falls in MS, there are still several critical issues to be resolved. One major area of concern relates to the lack of information regarding any association between falls and objective measures of balance and gait in this population. This is essential as the majority of falls occur during dynamic everyday activities. For example, upwards of 80% of falls occur during transfers and 60% during walking [3]. Furthermore, the majority of previous studies examining the relationship between walking, balance, and falls have been based on subjective clinical tests and self-report [1], [2], [3], [7]. Consequently, there has been little direct assessment of the relation between objective measures of gait and balance and previous falls history. Our understanding of the basis for the increased falls in this high risk group is further confounded by the fact that the association between previous fall history and clinical factors such as disability status (e.g. functional system scores of the expanded disability status scale: EDSS) has not been adequately addressed. Additionally, the association between falls and demographic factors such as gender and age is not clear [1], [6]. Consequently, the purpose of the current investigation was to determine the relation between fall history and clinical (EDSS and subcomponents), demographic (age, gender), mobility (walking speed, endurance, and coordination) and postural sway metrics in PwMS.

## Methods

### Ethics Statement

All procedures were approved by the local institutional review board of the Illinois College of Medicine at Peoria.

### Participants

The sample consisted of 52 individuals (44 females and 8 males) ranging in age from 30 to 73 years with MS who were recruited through three locally residing neurologists. To be included in the investigation, participants had a neurologist-confirmed diagnosis of MS, ability to walk independently or with a cane, crutch, or walker, comprehension of written and spoken English and had been relapse free for 30 days. Participants were divided into groups based on fall history (fallers and non-fallers); a fall was defined as an event where the participant unintentionally came to rest on the ground or a lower level [1]. Of the total number of persons assessed in this study, 23 had no history of falls over the past 12 months and 29 participants had a history of at least one fall within the same period.

### Procedures

All testing procedures were performed at a local clinic. Upon arrival to the clinic, the specific experimental procedures were explained to each participant. Prior to any evaluation, written informed consent was attained from all individuals. Specific demographic information was first attained from each participant. This was followed by specific experimental procedures which included a neurological exam (EDSS) performed by a neurologist (JHP), a multidimensional assessment of mobility, and a postural control assessment. The order of the testing was varied across participants. All procedures were approved by a local institutional review board.

### Measures

Demographic information collected included MS subtype, disease duration, age, assistive device use and gender. The number of falls in the past twelve month period was also recorded.

#### Neurologic Exam (EDSS)

The neurological exam allowed for the determination of each participant's EDSS score. The EDSS is a disability scale based on the function of eight different physiological systems. These include the pyramidal, cerebellar, brainstem, sensory, bowel/bladder, visual, mental and other^[8]^. Each subscale is graded from 0–5/6 with 0 denoting no disability and the upper scores (5 or 6) equating to maximum disability. Based on the specific functional scores for each system, an overall integrated score between 0 (no disability) and 10 (death) is determined for each person with MS^[8]^.

#### Mobility

A multidimensional assessment of mobility incorporated measures of walking speed, endurance, and coordination. Walking speed was quantified with the timed 25 foot walk (T25W) [9], walking endurance was assessed with the 6 minute walk (6MW) [10], and walking coordination was quantified with the six spot step test (6SST) [11] and the timed up and go (TUG) [12]. Additionally, spatio-temporal parameters related to gait (base of support and functional ambulation profile (FAP)) were collected utilizing a 26 foot electronic walkway (GAITRite™; CIR systems, Inc.) [13].

#### Balance

Standing postural control was assessed with posturography [14]. Posturography was accomplished by having participants stand barefoot with their feet shoulder width apart without aid (e.g. assistive device, etc) on a force platform (46.4×50.8 cm; AMTI, Inc). Participants completed a total of four 30 second trials. Two of the trials were completed with eyes open and two were performed with eyes closed. The platform records postural dynamics with 3 force components: the ML force (Fx), the anteroposterior force (Fy), and the vertical force (Fz) and 3 moment components: Mx, My, and Mz, which are the moments taken about the respective axes. The posture analysis was based on the motion of the center of pressure (COP). This component of postural data may be considered a reflection of the system's neuromuscular response to the imbalances of the body's center of gravity [15]. Based on previous work, the amount of postural sway was indexed with sway area (based on the 95% ellipse procedure) and velocity of postural sway along the ML and AP axes [14]. Due to unforeseen technical difficulties, postural sway metrics were only recorded for 41 of the 52 participants. This subsample was similar in demographic characteristics to the overall sample and was composed of 21 non-fallers and 20 fallers.

### Statistical analysis

All analyses were completed using SPSS version 17.0 (SPSS Inc, Chicago, IL) and significance was noted when *p*<0.05. Values are means ± standard deviation unless otherwise noted. Relative risk of assistive device use was calculated as the ratio of fallers who used an assistive to non-fallers who used an assistive device. Differences in dependent variables between groups were determined utilizing one-tailed independent samples *t*-tests based on the assumption that fallers would have worse walking and balance than non-fallers. Effect sizes based on a difference in mean scores were expressed as Cohen's *d* [16].

## Results

Demographic variables as a function of group are reported in table 1. In brief, participants were on average 53.1 years old (SD 11.3) and had MS for an average of 13.4 years (SD 9.4). Disability level of the participants ranged from an EDSS of 2.0 to 6.5 with a median of 4.0. Thirty two percent of the participants assessed utilized an assistive device during testing. Twenty-nine participants (55.8% of the sample) reported at least one fall in the previous 12 months with a further twenty-three of these 29 fallers (79% of fallers) reporting two or more falls in the same period. Typically, the individuals within the faller group were older, had longer disease duration, utilized an assistive device and had a greater level of disability than non-fallers (See Table 1). The differences between groups were moderate to large in magnitude based on effect sizes for age (*d* = −0.66) and MS duration (*d* = −0.80). The relative risk for a person who utilizes an assistive device to have fallen in the last 12 months was 3.84.

**Table 1:** Participant demographics as a function of group.

Variable	Non-Fallers	Fallers	p	*d/relative risk*
Age (years)	49.1 (12.1)	56.3 (9.7)	0.03	−0.66
MS Duration (years)	9.9 (6.3)	16.9 (10.6)	0.01	−0.80
Gender (female/male)	21/2	21/6	0.18	-
Assistive Device (%)	13%	50%	0.01	3.85
EDSS (median, IQR)	3.0 (2.0)	4.5 (2.5)	0.01	−0.81

### 

#### EDSS

An examination of EDSS subscales revealed that fallers had greater impairment in pyramidal, cerebellar, sensory, and bladder function than non-fallers (See table 2). There was an apparent difference in visual subscale between groups that did not reach traditional level of significance (*p* = 0.06). Overall, the differences between groups were moderate to large in magnitude based on effect sizes for cerebellar (*d* = −0.89), pyramidal (*d* = −0.53), sensory (*d* = −0.66) bladder/bowel (*d* = −0.44) and visual (*d* = −0.44).

**Table 2:** EDSS subscales as a function of group.

Variable	Overall	Non-Fallers	Fallers	t	p	d
Visual	1.0 (0.9)	0.8 (0.9)	1.2 (0.9)	−1.6	0.06	−0.44
Brainstem	1.4 (1.0)	1.3 (1.0)	1.4 (0.9)	−0.5	0.32	−0.11
Pyramidal	1.8 (0.7)	1.6 (0.8)	2.0 (0.7)	−1.9	0.02	−0.53
Cerebellar	1.5 (0.9)	1.0 (0.9)	1.8 (0.7)	−3.3	0.00	−0.89
Sensory	1.8 (0.7)	1.6 (0.7)	2.0 (0.5)	−2.4	0.01	−0.66
Bladder/Bowel	1.4 (0.9)	1.2 (0.9)	1.6 (0.9)	−1.8	0.04	−0.44
Mental	1.9 (0.6)	1.8 (0.7)	1.9 (0.5)	−0.9	0.20	−0.12

#### Mobility

Overall, individuals classified as fallers demonstrated diminished mobility scores for all assessments when compared to non-fallers (See table 3). Specifically, fallers had lower walking endurance (6MW distance) and walking coordination (TUG) compared to non-fallers. There was an apparent difference in walking speed (T25FW) between groups, but it did not reach traditional level of significance (*p* = 0.06). The differences between groups were of moderate magnitude based on effect sizes for 6MW (*d* = 0.61), TUG (*d* = −0.45) and T25FW (*d* = −0.46).

**Table 3:** Mobility metrics as a function of group.

Variable	Overall	Non-Fallers	Fallers	t	p	*d*
T25FW (s)	6.4 (2.6)	5.8 (2.9)	7.0 (2.3)	−1.6	0.06	−0.46
6MW (feet)	1398 (410)	1533 (454)	1288 (341)	2.2	0.02	0.61
TUG (s)	8.9 (4.2)	7.8 (4.7)	9.7 (3.7)	−1.7	0.04	−0.45
6SST (s)	10.3 (5.2)	9.2 (5.6)	11.2 (4.8)	−1.4	0.09	−0.38
FAP	90.7 (12.1)	92.0 (2.5)	89.5 (2.5)	0.7	0.24	0.10
Gait Speed (cm/s)	106.7 (27.5)	112.8 (28.5)	101.3 (25.9)	1.5	0.07	0.42
Base of Support (cm)	12.4 (5.1)	12.6 (6.0)	12.2 (4.2)	0.3	0.41	0.07

Note: T25FW = timed 25 foot walk; 6MW = 6 minute walk; TUG = Time up and go; 6SST = six spot step test; FAP = functional ambulation profile.

#### Balance

Persons classified as fallers exhibited increased sway velocity in the ML direction with eyes open, and greater overall sway area (See table 4). In addition, under eyes closed (EC) conditions, fallers had greater sway velocity in the ML and AP directions, compared to non-fallers. The differences between groups were moderate to large based on effect sizes for ML sway velocity (*d* = −0.49), sway area_EC_ (*d* = −0.62) and sway velocity_EC_ in the AP and ML axis (*d* = −0.64 and −0.84, respectively).

**Table 4:** Balance metrics as a function of group.

Variable	Overall	Non-Fallers	Fallers	t	p	*d*
Sway Area (cm^2^)	147.0 (34.4)	139.1 (29.6)	154.6 (30.0)	−0.4	0.36	−0.52
Sway Area_EC_ (cm^2^)	330.3 (413.7)	207.2 (220.5)	453.4 (520.1)	2.0	0.02	−0.62
Sway Velocity (AP)	7.4 (4.0)	7.2 (4.3)	7.6 (3.7)	−0.4	0.36	−0.10
Sway Velocity (ML)	9.7 (4.5)	8.6 (5.1)	10.8 (3.7)	−1.6	0.05	−0.49
Sway Velocity_EC_ (AP)	10.8 (6.3)	8.8 (4.6)	12.7 (7.2)	−2.1	0.04	−0.64
Sway Velocity_EC_ (ML)	17.8 (10.2)	13.8 (8.4)	21.8 (10.5)	−2.8	0.01	−0.84

AP = anterior-posterior; ML = mediolateral; EC = eyes closed.

## Discussion

The purpose of the current investigation was to assess the demographic, clinical, and mobility factors related to fall history in persons with MS. Overall, those individuals classified as fallers tended to be older, had a greater prevalence of assistive devices use, increased disability, decreased walking endurance and coordination, greater perceived walking impairment and poorer balance than those individuals classified as non-fallers. Additionally, fallers had greater impairment in cerebellar, pyramidal, sensory, bladder/bowel and vision subscales of the EDSS. The current findings serve to bridge gaps in the extant literature concerning demographic, clinical, and mobility factors related to previous fall history in PwMS.

Within the current sample, a sizeable majority of the participants (55.8%) reported at least one fall in the previous 12 month period with a further 79% of these persons reporting two or more falls. This pattern of results in consistent with previous reports [1], [2], [3], [7]. For instance, Finlayson and colleagues [1] reported a fall occurrence of 52.2% over 6 months in a sample of 1089 PwMS while Matsuda and colleagues [3] reported a fall incidence of 58.2% in a sample of 265 PwMS over 6 months. Nilsagard [2] report a fall incidence of 63% over 9 months in 76 PwMS and that ∼73% of the fallers were recurrent fallers. The finding that over half of MS individuals suffered a fall is a major concern for overall health status and quality of life, especially when contrasted with reports stating that only one third (33%) of community dwelling healthy adults over 65 years of age fall in a 12 month period and 10% are recurrent fallers [17]. Together this pattern of results highlights that this population group is at a dramatically higher risk of falling that healthy individuals of a similar age.

### Demographics Factors related to Falls

It has been suggested that older adults with MS should be at greater risk for falls due to the combination of age- and MS-related changes to physiological systems involved in balance and postural control [6]. However, the empirical data supporting this claim is minimal [1], [2], [3], [7]. Indeed, to our knowledge, the current investigation is the first data set to report a strong association between age and previous falls history for PwMS. A potential explanation of this novel finding is that age has been examined as a non-continuous variable [1] since those investigations which did not find an association between age and falls in PwMS had a relatively young sample [7]. Given that falls risk increases dramatically for individuals over 65 years of age, there is a strong possibility that any adverse effects of this disease process on postural control would be more likely to have an impact for the older population with MS. Consequently, it would be the combination of age and disease which would lead to the greatest risk of falls, and not the singular effects of these factors. Further work is needed to fully understand the association between falls and age in PwMS.

Consistent with previous studies, those persons classified as fallers reported greater use of assistive device during locomotion [2], [3], [7] than non-fallers. It is possible that assistive device use is simply a proxy of walking impairment or balance difficulties. An alternative possibility is that assistive device use can actually have negative consequences for postural control since it minimizes the intrinsic ability to recover balance as well as increase energy demands of walking [18].

### Clinical Factors and Falls

The results of the current study support the general view that increased level of disability for individuals with MS typically translates to an increased likelihood of suffering a previous fall [1], [2]. Fallers had a greater level of disability as indexed by the neurologic exam (EDSS) than non-fallers. Recently, it has been reported that PwMS who have fallen in the last six months have increased lesions in the cerebellum and brainstem compared to non-fallers [19]. While those persons classified as fallers within the current investigation had greater impairment in cerebellar, sensory, pyramidal, vision and bladder/bowel function there was no difference in brainstem functional scores between groups, as indexed by a neurologist-derived EDSS score. Given that there is a significant contribution of visuomotor processing to balance impairment in PwMS [20], [21], it is not surprising that fallers had worse visual function. Congruent with this observation, Kasser and colleagues [22] recently found that visually dependent sway was predictive of future falls in women with MS.

Additionally, given the contribution proprioception has for optimal postural control, the strong link between falls and sensory dysfunction is not to be unexpected l [23]. This relation was supported by the increase in postural sway metrics for fallers observed under the eyes closed conditions (i.e. when proprioceptive information and other non-visual sensory input are utilized more). The association between pyramidal dysfunction and falls could be indicative of the contribution of spasticity to falls in PwMS [2], whereas the association between cerebellar function and falls is consistent with the contribution of balance to falls in PwMS [7], [19].

Another factor which has been linked with increased falls risk is bladder or bowel problems [1], [3]. Incontinence has been associated with falls in older adults and it has been suggested that frequent and urgent trips to the bathroom place individuals at greater risk of falls [24]. It is not clear why brainstem dysfunction was not related to falls in the current sample [19]. It is possible that lack of an association in the current sample results from the limits of the EDSS functional system scoring.

### Mobility, Balance and Falls

Despite the adverse impact falls have on quality of life and general function in PwMS, there have been relatively few investigations of those specific risk factors related to falls for this high risk population. While a number of “generic” falls risk tests have been developed for healthy community dwelling elderly population, these are less appropriate for differentiating falls risk in people with MS as their balance and gait deficits are unique and more pronounced. While a handful of extant investigations have been designed to assess this concern, their findings have largely been inconsistent [1], [2], [7]. Further limitations of these previous reports is their reliance on self-reports [1], subjective measures [7] and/or a limited number of walking and balance tests [2]. A major strength of the current investigation was the use of a battery of tests spanning multiple dimensions of balance and postural control. Such a multi-dimensional battery has a greater potential to tease out and identify differences and changes in mobility. Indeed, the finding that walking endurance and coordination are distinctly different between MS persons with a history of falling and those classified as non-fallers was based upon this test battery.

The contribution of walking endurance to fall history observed here raises the possibility that fatigue and tiredness contributes to falls in PwMS. This notion is further bolstered by reports that self-reported fatigue is related to fall incidence in PwMS [2], [3]. A potential implication of this observation is that fall prevention interventions in PwMS should focus on walking endurance and/or reducing the impact of fatigue.

It has been previously reported that balance impairment can be associated with increased falls risk in PwMS [1], [2], [7]. However, as previously mentioned, these reports have utilized either self-reports of overall balance ability [1] or subjective measures such as the Berg Balance scale [2], [7]. One major advantage of utilizing balance plates for assessing posture is their ability to objectively quantify the degree of postural motion in each plane. This is particularly relevant since even small increases in sway in the medio-lateral direction has been associated with falls in both older adults [25], [26] and those with MS [19]. Indeed, the results of the current study demonstrated that differences in COP sway velocity in both the AP and ML directions were seen between fallers and non-fallers.

One possible contributing factor to the increased falls risk within this population group relates to the increased muscle tone seen with spasticity. In a prospective investigation, Nilsagard [2] found that for every incremental increase in spasticity, the likelihood falling more than doubles. Although, the current investigation did not specifically quantify spasticity, the greater amount of dysfunction in the pyramidal subscale of the EDSS in fallers and the fact that pyramidal function can be linked to spasticity, indirectly suggests that spasticity could be a contributing factor for the increased falls risk reported in the current investigation. Additionally, there is growing evidence that elevated spasticity can be associated with diminished performance on T25FW [27], 6MW [27], [28], TUG [27] and impaired postural control [14], [27]. Specifically, elevated spasticity has been found to related to mediolateral sway velocity in PwMS [14]. Consequently, it is possible that the contribution of these mobility tests to falls risk is being driven in part by the contribution of spasticity to fall history and risk. This notion should be tested in future research.

### Future research and Limitations

Despite the prevalence and adverse impact of falls in PwMS, there have been few investigations of interventions aimed at minimizing fall risk. There is evidence that physiological risk factors can be minimized with exercise training [29], [30], [31], [32] and an exercise intervention may translate into a decrease in fall risk as documented in community-dwelling older adults [33]. Further work is needed to determine if similar interventions will reduce falls in PwMS.

There are several limitations within the current investigation. One limitation is the relatively small sample size (n = 52). Additionally, caution should be used when relaying on recall measures in populations with documented cognitive impairment. However, there is data to suggest that fall recall in PwMS is relatively accurate [2].

### Conclusion

The current observations suggest that PwMS who are older, walk slower, and have worse balance and decreased walking endurance are at a greater risk of falls. It is suggested that individuals who meet these criteria be carefully monitored for future falls. Future research is needed to understand the consistency of fall risk factors across the disability spectrum in PwMS and to determine a prospective model of falls in PwMS. Additionally, the utility of interventions aimed at reducing falls and fall risk in PwMS needs to be established.

## References

[1] Finlayson ML, Peterson EW, Cho CC (2006). Risk factors for falling among people aged 45 to 90 years with multiple sclerosis.. Arch Phys Med Rehabil.

[2] Nilsagard Y, Lundholm C, Denison E, Gunnarsson LG (2009). Predicting accidental falls in people with multiple sclerosis – a longitudinal study.. Clin Rehabil.

[3] Matsuda PN, Shumway-Cook A, Bamer AM, Johnson SL, Amtmann D (2011). Falls in multiple sclerosis.. PM R.

[4] Cameron MH, Poel AJ, Haselkorn JK, Linke A, Bourdette D (2011). Falls requiring medical attention among veterans with multiple sclerosis: a cohort study.. J Rehabil Res Dev.

[5] Peterson EW, Cho CC, von Koch L, Finlayson ML (2008). Injurious falls among middle aged and older adults with multiple sclerosis.. Arch Phys Med Rehabil.

[6] Finlayson ML, Peterson EW (2010). Falls, aging, and disability.. Phys Med Rehabil Clin N Am.

[7] Cattaneo D, De Nuzzo C, Fascia T, Macalli M, Pisoni I (2002). Risks of falls in subjects with multiple sclerosis.. Arch Phys Med Rehabil.

[8] Kurtzke JF (1983). Rating neurologic impairment in multiple sclerosis: an expanded disability status scale (EDSS).. Neurology.

[9] Kaufman M, Moyer D, Norton J (2000). The significant change for the Timed 25-foot Walk in the multiple sclerosis functional composite.. Mult Scler.

[10] Goldman MD, Marrie RA, Cohen JA (2008). Evaluation of the six-minute walk in multiple sclerosis subjects and healthy controls.. Mult Scler.

[11] Nieuwenhuis MM, Van Tongeren H, Sorensen PS, Ravnborg M (2006). The six spot step test: a new measurement for walking ability in multiple sclerosis.. Mult Scler.

[12] Nilsagard Y, Lundholm C, Gunnarsson LG, Dcnison E (2007). Clinical relevance using timed walk tests and ‘timed up and go’ testing in persons with multiple sclerosis.. Physiother Res Int.

[13] Sosnoff JJ, Weikert M, Dlugonski D, Smith DC, Motl RW (2011). Quantifying gait impairment in multiple sclerosis using GAITRite technology.. Gait & Posture.

[14] Sosnoff JJ, Shin S, Motl RW (2010). Multiple sclerosis and postural control: the role of spasticity.. Arch Phys Med Rehabil.

[15] Winter DA (1993). Biomechanics and motor control of human movement.

[16] Cohen J (1988). Statistical power analysis for the behavioral sciences.

[17] Lord SR, Ward JA, Williams P, Anstey KJ (1993). An epidemiological study of falls in older community-dwelling women: the Randwick falls and fractures study.. Aust J Public Health.

[18] Bateni H, Maki BE (2005). Assistive devices for balance and mobility: benefits, demands, and adverse consequences.. Arch Phys Med Rehabil.

[19] Prosperini L, Kouleridou A, Petsas N, Leonardi L, Tona F (2011). The relationship between infratentorial lesions, balance deficit and accidental falls in multiple sclerosis.. J Neurol Sci.

[20] Cattaneo D, Jonsdottir J (2009). Sensory impairments in quiet standing in subjects with multiple sclerosis.. Mult Scler.

[21] Van Emmerik RE, Remelius JG, Johnson MB, Chung LH, Kent-Braun JA (2010). Postural control in women with multiple sclerosis: effects of task, vision and symptomatic fatigue.. Gait & Posture.

[22] Kasser SL, Jacobs JV, Foley JT, Cardinal BJ, Maddalozzo GF (2011). A Prospective Evaluation of Balance, Gait, and Strength to Predict Falling in Women With Multiple Sclerosis.. Arch Phys Med Rehabil.

[23] Cameron MH, Horak FB, Herndon RR, Bourdette D (2008). Imbalance in multiple sclerosis: a result of slowed spinal somatosensory conduction.. Somatosens Mot Res.

[24] Brown JS, Vittinghoff E, Wyman JF, Stone KL, Nevitt MC (2000). Urinary incontinence: does it increase risk for falls and fractures? Study of Osteoporotic Fractures Research Group.. J Am Geriatr Soc.

[25] Maki BE, Holliday PJ, Topper AK (1994). A prospective study of postural balance and risk of falling in an ambulatory and independent elderly population.. J Gerontol.

[26] Piirtola M, Era P (2006). Force platform measurements as predictors of falls among older people - a review.. Gerontology.

[27] Sosnoff J, Gappmeier E, Frame A, Motl RW (in press). Spasticity, Mobility and Balance in Persons with MS.. Journal of Neurological Physical Therapy.

[28] Kuspinar A, Andersen RE, Teng SY, Asano M, Mayo NE (2010). Predicting exercise capacity through submaximal fitness tests in persons with multiple sclerosis.. Arch Phys Med Rehabil.

[29] Coote S, Garrett M, Hogan N, Larkin A, Saunders J (2009). Getting the balance right: a randomised controlled trial of physiotherapy and Exercise Interventions for ambulatory people with multiple sclerosis.. BMC Neurol.

[30] Cattaneo D, Jonsdottir J, Zocchi M, Regola A (2007). Effects of balance exercises on people with multiple sclerosis: a pilot study.. Clin Rehabil.

[31] Morrison S, Colberg SR, Mariano M, Parson HK, Vinik AI (2010). Balance training reduces falls risk in older individuals with type 2 diabetes.. Diabetes Care.

[32] Hebert JR, Corboy JR, Manago MM, Schenkman M (2011). Effects of Vestibular Rehabilitation on Multiple Sclerosis-Related Fatigue and Upright Postural Control: A Randomized Controlled Trial.. Phys Ther.

[33] Gillespie L, Handoll H (2009). Prevention of falls and fall-related injuries in older people.. Inj Prev.

